# Primary localized rectal/pararectal gastrointestinal stromal tumors: results of surgical and multimodal therapy from the French Sarcoma group

**DOI:** 10.1186/1471-2407-14-156

**Published:** 2014-03-05

**Authors:** Thanh-Khoa Huynh, Pierre Meeus, Philippe Cassier, Olivier Bouché, Sophie Lardière-Deguelte, Antoine Adenis, Thierry André, Julien Mancini, Olivier Collard, Michael Montemurro, Emmanuelle Bompas, Maria Rios, Nicolas Isambert, Didier Cupissol, Jean-Yves Blay, Florence Duffaud

**Affiliations:** 1 Service d’Oncologie Médicale, CHU Timone, AP-HM, Marseille, and Aix-Marseille Université, Marseille, France; 2 Service de Chirurgie, Centre Léon Bérard, Lyon, France; 3 Service d’Oncologie Médicale, Centre Léon Bérard, Lyon, France; 4 Service d’Oncologie Digestive, CHU Robert Debré, Reims, France; 5 Service de Chirurgie, CHU Robert Debré, Reims, France; 6 Service d’Oncologie Médicale, Centre Oscar Lambret, Lille, France; 7 Service d’Oncologie Médicale, Hôpital St Antoine, Assistance Publique–Hôpitaux de Paris and Université Pierre & Marie Curie (UPMC), Paris, France; 8 Service de Santé Publique et d’Information Médicale, Unité de Biostatistique, CHU Timone, APHM, Marseille, and Aix-Marseille Université, Marseille, France; 9 Service d’Oncologie Médicale, CLCC, Institut de Cancérologie Lucien Neuwirth, Saint-Etienne, France; 10 Centre Pluridisciplinaire d’Oncologie, CHUV Lausanne, Lausanne, Suisse; 11 Service d’Oncologie médicale, Centre René Gauduchau, Nantes, France; 12 Service d’Oncologie Médicale, Centre Alexis Vautrin, Vandoeuvre les, Nancy, France; 13 Service d’Oncologie Médicale, Centre George François Leclerc, Dijon, France; 14 Service d’Oncologie Médicale, Centre Valdorelle, Montpellier, France; 15 Service d’Oncologie Médicale Adulte, CHU Timone, AP-HM, 264 rue Saint Pierre, 13385 Marseille, France

## Abstract

**Background:**

Rectal and pararectal gastrointestinal stromal tumors (GISTs) are rare. The optimal management strategy for primary localized GISTs remains poorly defined.

**Methods:**

We conducted a retrospective analysis of 41 patients with localized rectal or pararectal GISTs treated between 1991 and 2011 in 13 French Sarcoma Group centers.

**Results:**

Of 12 patients who received preoperative imatinib therapy for a median duration of 7 (2-12) months, 8 experienced a partial response, 3 had stable disease, and 1 had a complete response. Thirty and 11 patients underwent function-sparing conservative surgery and abdominoperineal resection, respectively. Tumor resections were mostly R0 and R1 in 35 patients. Tumor rupture occurred in 12 patients. Eleven patients received postoperative imatinib with a median follow-up of 59 (2.4-186) months. The median time to disease relapse was 36 (9.8-62) months. The 5-year overall survival rate was 86.5%. Twenty patients developed local recurrence after surgery alone, two developed recurrence after resection combined with preoperative and/or postoperative imatinib, and eight developed metastases. In univariate analysis, the mitotic index (≤5) and tumor size (≤5 cm) were associated with a significantly decreased risk of local relapse. Perioperative imatinib was associated with a significantly reduced risk of overall relapse and local relapse.

**Conclusions:**

Perioperative imatinib therapy was associated with improved disease-free survival. Preoperative imatinib was effective. Tumor shrinkage has a clear benefit for local excision in terms of feasibility and function preservation. Given the complexity of rectal GISTs, referral of patients with this rare disease to expert centers to undergo a multidisciplinary approach is recommended.

## Background

Gastrointestinal stromal tumors (GISTs) are rare but nonetheless represent the most common mesenchymal tumors of the gastrointestinal tract. The majority of GISTs arise in the stomach and small intestine [1], with an estimated annual incidence of 11 to 14.5 per million [2,3]. Rectal/pararectal GISTs are very rare and represent only 3 to 5% of all GISTs. Their incidence is estimated at 0.45 per million per year [4]. Few series of rectal GISTs have been reported in the literature, and the available reports are limited to a small number of cases [5-7].

Due to the rarity of rectal GISTs and the limited number of published studies, there is a paucity of data on how to optimally handle rectal GISTs. There is a tendency to treat rectal/pararectal GISTs as other GISTs, particularly as gastric GISTs. Management typically involves *en bloc* resection of the tumor, which avoids tumor rupture and obtains clear margins; there is no need for lymphadenectomy because lymphatic metastases are exceedingly rare. Nevertheless, rectal/pararectal GISTs have a high risk of recurrence (local recurrence or metastasis), ranging from 55% for tumors of >5 cm with a mitotic index (MI) of ≤5/50 HPF to 85% for tumors with an MI of >5/50 HPF regardless of size [8].

Surgery remains the only curative treatment for GISTs. However, because of the specific location of rectal GISTs, surgery is technically difficult and often extensive, possibly involving abdominoperineal or multivisceral resections, and raises the problem of sphincter preservation. Extensive surgery may result in considerable functional morbidity based on the tumor size, exact location of the tumor, and relationship of the tumor with vital pelvic structures (i.e., bladder, pelvic nerves, and anal sphincters). Therefore, the ability to shrink such a tumor in a safe and reliable manner is crucial to facilitate the performance of function-sparing surgical resection of disease.

Imatinib is a selective receptor tyrosine kinase inhibitor of the KIT and PDGFR-α receptor tyrosine kinases, which are pathophysiological drivers of GISTs. Imatinib is approved worldwide as a first-line systemic treatment for KIT-positive unresectable and/or metastatic GISTs and has revolutionized their treatment [9]. Since 2005, several case reports and small series regarding use of preoperative “neoadjuvant” imatinib treatment for rectal GISTs have been published [10-14], and some larger series have been more recently published [15,16]. These studies have shown that significant downstaging can be achieved with this targeted therapy, thus allowing conservative surgical procedures to be performed. More recently, imatinib treatment has been considered in the adjuvant setting to lower the risk of relapse [17]. Published reports of patients with rectal GISTs treated since the approval of imatinib, which has dramatically changed the treatment options and prognosis for GISTs, are rare [15,16].

We conducted a 19-year retrospective analysis of rectal/pararectal GISTs with the aim of reviewing the clinicopathological characteristics, diagnostic and treatment approaches, choice of surgical procedure, perioperative use of imatinib, patterns of failure, and early and long-term results including overall survival and event-free survival.

### Methods

We collected data of adult patients with rectal/pararectal GISTs treated from November 1991 to March 2011 in 12 French Sarcoma Group institutions and the Centre Hospitalier Universitaire Vaudois (CHUV) Lausanne. A standard data file was created to retrieve information on patient characteristics (gender, age at diagnosis, Eastern Cooperative Oncology Group [ECOG] performance status, and initial clinical symptoms), clinicopathological tumor characteristics, treatment approaches including surgical management and medical treatment (neoadjuvant and/or adjuvant imatinib), and patterns of failure (local recurrence or distant metastasis). Follow-up information was obtained during outpatient visits.

The diagnosis of GIST was confirmed by an experienced local pathologist using morphology and immunohistochemical staining for KIT (CD117) and CD34. Seven GISTs diagnosed as leiomyosarcoma before the year 2000 were reclassified as GISTs at the time of recurrence. Tumors were classified using standard risk assessment criteria using both the classification proposed by Fletcher (NIH consensus risk) [18], which is based on tumor size and number of mitoses, and the classification proposed by Miettinen [19], which is based on the MI, tumor size, and tumor site.

### Ethics statement

This was a retrospective minimal-risk review, and all patients consented to the use of their standard clinical data. Our institutional review board exempts such minimal-risk survey studies from requiring institutional review board approval according to French laws. The board of directors of the French Sarcoma Group approved the study.

### Statistical analysis

Continuous variables are expressed as median (range), and categorical variables are expressed as percentages. We retrospectively analyzed all prognostic factors, including patient characteristics (sex, age, ECOG performance status), tumor characteristics (tumor site in rectum, tumor size, KIT, CD34, MI/50 HPF, NIH categories, Miettinen categories, necrosis, histological subtype, and mutational status), surgical management (type of procedure, radical vs conservative surgery, margins [R0 vs R1 vs R2 or R0 vs R1-R2 or R0-R1 vs R2], tumor rupture, surgery-related complications), and medical treatment (imatinib treatment group vs non-imatinib treatment group). Fisher’s exact test was used to compare percentages. Survival curves were plotted using the Kaplan–Meier method and compared with the log-rank test. Overall survival was calculated based on the interval from diagnosis to patient’s death or last follow-up. Local relapse-free survival was defined as the interval between diagnosis and any subsequent occurrence of a tumor in the same location. Event-free survival was defined as the interval between diagnosis and local relapse and/or distant metastasis. All statistical tests were two-sided, and the threshold for statistical significance was set at p = 0.05. Analyses were performed with SPSS software, version 17.0 (SPSS Inc., IL, USA).

## Results

### Patient and disease characteristics

Patient and tumor characteristics are described in Table 1.

**Table 1:** Patient and tumor characteristics

**Characteristics**	**n = 41**
Gender	
Male	29 (71%)
Female	12 (29%)
Age (years)	
Median (min-max)	60 (33-82)
ECOG	
0/1	16/15 (98%)
Symptoms-Clinical manifestations	
Abdominal pain	9 (22%)
Constipation	4 (10%)
Rectal bleeding	5 (12%)
Accidental discovery	9 (22%)
Others (genito urinary, pelvic heaviness)	6 (15%)
Unknown	8 (19%)
Tumor characteristics	
Tumor location	
Rectum (Middle third/Lower third)	9/22 (24/50%)
Pararectal space	7 (17%)
Unspecified	3 (9%)
Tumor size (mm)	
median (min-max)	62 (6-130)
Evaluated by:	
Abdominopelvic CT Scan	21 (51%)
MRI	18 (44%)
Endosonography	23 (56%)
Unknown	6 (14%)
Histology/Genotype/risk	
CD117+	34 (83%)
CD34+	
Mitotic index	
≤ 5/50 HPF	11 (27%)
> 5/50 HPF	21 (51%)
Unknown	9 (22%)
Mutation status (done on 12 cases)	*KIT* Exon 11 mutation in 10/12
	*KIT* Exon 9 mutation in 1/12
	*KIT* Exon 17 mutation in 1/12
	None PDGFRA mutation
**NIH risk categories**	
Very low risk	3 (7%)
Low risk	3 (7%)
Intermediate risk	6 (14%)
High risk	18 (42%)
**Miettinen risk categories**	
None risk	3 (7%)
Low risk	4 (10%)
High risk	23 (56%)
Insufficient data (unclassified)	11 (26%)

During the period from November 1991 to March 2011, a total of 41 adult patients with localized rectal/pararectal GISTs were included in this review, including 4 patients reported in a separate therapeutic trial known as the French Sarcoma Group BFR 14 study [20]. The median age at diagnosis was 60 years (range, 33-82 years). The staging of the primary tumor included abdominopelvic computed tomography (CT) in 21 patients (51%), pelvic magnetic resonance imaging (MRI) in 18 patients (44%), endorectal ultrasonography in 23 patients (56%), and positron emission tomography–CT in 6 patients (15%).

The median tumor size was 62 mm (range, 6-130 mm). GISTs were mostly located in the middle third (9 patients, 24%) and lower third (22 patients, 50%) of the rectum. Only seven GISTs (17%) were located in pararectal spaces (presacral space, ischiorectal fossa, rectovaginal space, and rectoprostatic space). Histologically, the tumors were predominantly spindle-cell type (n = 31, 76%). Immunohistochemically, 83% of the tumors were KIT (CD117)-positive, and 78% were CD34-positive. The mutational status was determined in only 12 cases and revealed *KIT* exon 11 mutations in 10 cases.

### Treatment

Treatment modalities (surgery and imatinib therapy) are presented in Table 2.

**Table 2:** Results of treatment

**Characteristic**	**n = 41 localized tumors**
Quality of surgery	
Resection	
R0	22 (53%)
R1	13 (32%)
R2	4 (10%)
Tumor rupture	
Yes	9 (22%)
No	29 (70%)
Primary surgery/Post imatinib surgery	29/12
Post-operative complications	7 (17%)
Anastomotic leakage of coloanal anastomosis	2
Small bowel fistula with death	1
Pelvic peritonitis	1
Pararectal abscess and anorectal fistula	1
Occlusion	1
Fever	1
Imatinib (IM) therapy group (400 mg/day)	16 (37.5%)
Preoperative IM	12 (30%)
No neoadjuvant IM group (immediate surgery)	29 (70%)
Post operative IM	11 (27%)
from « neoadjuvant group »	7
from « immediate surgery group »	4
Median duration preoperative IM (month)	7 (2-12)
Median duration post operative IM (month)	7 (2-41)
Efficacy of preoperative IM therapy	12
Partial response	8
Complete response	1
Stable disease/minor response	3

#### Treatment strategy

All patients underwent tumor resection. Twenty-five patients underwent surgery only. Imatinib was not given either because of the small tumor size or because it was not available at the time of diagnosis. A total of 16 patients underwent operations and received preoperative (n = 5), postoperative (n = 4), or preoperative and postoperative (n = 7) receptor tyrosine kinase inhibitor therapy with imatinib. Twenty-nine (70%) patients underwent surgery as the initial treatment, but 12 were treated with imatinib preoperatively because of the tumor size.

#### Surgery

All patients underwent surgery. Different types of surgical procedures were performed. Local removal of the tumor was performed in 30 of 41 patients and involved anterior resection with coloanal or sus-anal anastomosis in 12 (29%) patients, transanal/trans-sacral/transvaginal resection in 7 (17%) patients, endoanal/endovaginal excision in 6 (14.6%) patients, and unspecified resection in 5 (12%) patients, resulting in 14 R0, 10 R1, and 4 R2 resections of 30 conservative surgeries and 2 unspecified resections. Abdominoperineal resection was performed in 11 of 41 (27%) patients, resulting in 8 of 11 R0, 3 of 11 R1, and no R2 resections. In proportion, sphincter-sparing surgery was performed in 61% of patients, and abdominoperineal resection was performed in 27% of patients. Abdominoperineal resection was more likely to result in negative margins than local surgery (8 of 11 vs 14 of 30, respectively; p < 0.05).

Seven patients (17%) experienced complications related to surgery: infection (n = 4), occlusion (n = 1), and disunity of the coloanal anastomosis (n = 2). Complications were fatal in one patient who died of a small bowel fistula. Tumor rupture occurred during surgery in nine patients (22%).

#### Preoperative imatinib therapy

Among the 41 patients, 12 (30%) received preoperative imatinib at 400 mg daily before surgical resection, with a median preoperative treatment period of 7 months (range, 2-12 months). All tumors except two were in the inferior third of the rectum. The tumor response was assessed every 2 to 3 months by CT and/or MRI. Of the 12 patients, 8 showed a partial response according to Response Evaluation Criteria in Solid Tumors (RECIST), 1 had a complete response, and 3 had stable disease or a minor response.

Two patients underwent abdominoperineal resection despite a partial response after imatinib therapy; all had negative margins and no postoperative complications. Ten patients underwent a sphincter-sparing surgery; all except one had negative margins (R0), and four of them had postoperative complications.

### Postoperative imatinib therapy

According to their estimated risk of relapse, 11 (27%) patients were treated with imatinib postoperatively at 400 mg/day for a median duration of 7 months (range, 2-41 months). Seven after surgery performed after preoperative imatinib therapy and four after surgery only.

### Patient outcomes (Table 3)

**Table 3:** Outcome

**Characteristics**	**n = 41 localized tumors**
Follow-up (month)	
Median	59
min	2.4
max	186
Prognosis	
Death	9
Local relapse	20 (49%)
Metastases	8
Local relapse	
In the « non imatinib treatment group »	18 (72%)
In the « imatinib treatment group »	2 (16%)
6/11 patients (54.5%) who underwent abdominoperineal resection (exclusively in « non IM group)	
Metastases	
In the « non imatinib treatment group »	6
In the « imatinib treatment group »	2
Local relapse-free survival at 3 years	60.2% [CI95% = 45.8-79.1]
Local relapse-free survival at 5 years	42.1% [CI95% = 26.8-66.3]
DFS at 3 years	53.9% [CI95% = 39.3-73.8%]
DFS at 5 years	34.6% [CI95% = 19.9-60.2%]
OS at 3 years	97.5% [CI95% = 92.8-100%]
OS at 5 years	86.5% [CI95% = 74.9-100%]

#### Recurrence

Local recurrence occurred in 20 (49%) patients, including 18 (72%) in the non-imatinib treatment group and only 2 (16%) in the imatinib treatment group (p < 0.001). The median follow-up duration in the imatinib treatment group was 39 months (range, 5-81 months), and that in the non-imatinib treatment group was 68 months (range, 2-186 months).

Among the 20 local recurrences, 6 (54.6%) developed in the 11 patients who underwent abdominoperineal resection (exclusively in the non-imatinib treatment group), and 14 (46%) developed among the 30 patients who underwent local resection (2 who received imatinib and 12 who did not receive imatinib). Furthermore, these local recurrences developed in 8 of 22 patients after R0 resection, 9 of 13 patients after R1 resection, and 3 of 4 patients after R2 resection. Eight of 41 patients (20%) developed distant metastases: 6 (24%) in the non-imatinib treatment group and 2 (13%) in the imatinib treatment group (p = 0.448).

#### Survival analysis and prognostic factors

**Figure 1:**
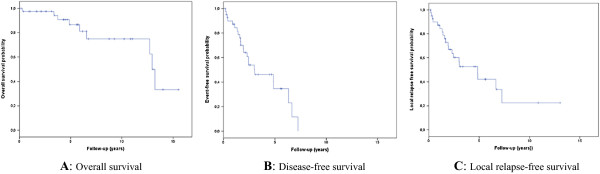
Survival curves.

The median overall follow-up period was 59 months (range, 2.4-186 months). Nine patients died during the follow-up period. The 3-and 5-year overall survival rates of patients with localized rectal/pararectal GISTs were 97.5 and 86.5%, respectively (Figure 1A). The median event-free survival period was 36 months (range, 9.8-62 months), with 3-and 5-year event-free survival rates of 53.9 and 34.6%, respectively (Figure 1B). The median local relapse-free survival period was 58 months (range, 29-86 months), with 3-and 5-year local relapse-free survival rates of 60.2 and 42.1%, respectively (Figure 1C).

**Figure 2:**
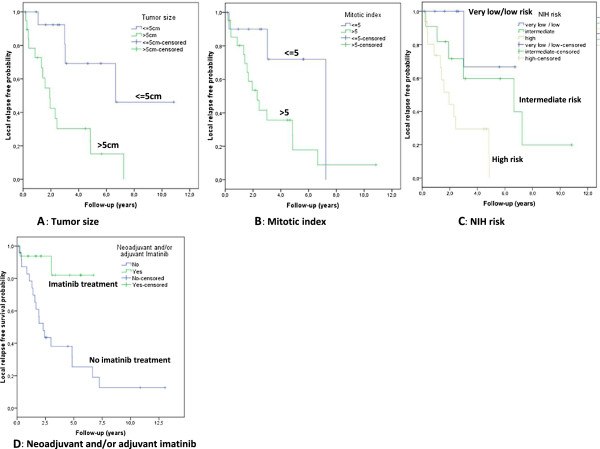
Factor associated with local relapsed-free survival.

In the univariate analysis, only tumor size (p = 0.004) (Figure 2A), mitotic count (p = 0.048) (Figure 2B), NIH risk (p = 0.023) (Figure 2C), and imatinib treatment (p = 0.006) (Figure 2D) were predictive of local relapse.

A tumor size of larger than 5 cm and an MI of higher than 5/50 HPF increased the risk of local recurrence, as expected with these known prognostic factors [16]. A low MI (≤5/50 HPF) (p = 0.008) and imatinib treatment (p = 0.011) were associated with a significantly lower risk of overall relapse (local recurrence and distant metastasis), but had no impact on overall survival. Sex, age, NIH risk, Miettinen risk categories, tumor margins, tumor rupture, and tumor local control had no influence on event-free survival or overall survival in the univariate analysis. In the multivariate analysis, we could not demonstrate whether tumor size, MI, or NIH risk were independent factors.

Preoperative and/or postoperative imatinib treatment significantly reduced the risk of overall relapse (p = 0.011) and local relapse (p = 0.006) (Figure 2D), with a significant impact on disease-free survival but no demonstrable impact on overall survival.

## Discussion

GISTs are unusual tumors overall, while rectal GISTs are a particularly rare subtype, representing less than 5% of all GISTs. Few data are available on the presentation, management, and survival of patients with primary rectal GISTs.

Our retrospective study of 41 localized rectal GISTs over the last 20 years is, to our knowledge, one of the largest series currently reported in the literature. The two most recent and large series on primary rectal GISTs reported 39 cases from 2 referral departments for sarcomas/GIST surgeries over the last 8 years [16] and 32 cases from 6 surgical oncology and medical oncology departments in the Netherlands over the last 21 years [15].

Patient demographics showed a male predominance (71%), as already suggested in other series on primary rectal GISTs [8,15,16,21], although most large epidemiological studies of GISTs overall have shown a well-balanced sex ratio with respect to incidence [2,22]. The presentation and symptoms of these 41 rectal GISTs were not different from those commonly found for other rectal tumors despite the fact that the clinical presentation was sometimes misleading, with rectal GISTs causing genitourinary and gynecological symptoms because of compression or invasion of adjacent pelvic organs. Therefore, we agree with other authors [6,16,23] that pelvic/rectal GISTs should be integrated into the framework of differential diagnoses along with other submucosal rectal and pelvic tumors.

The mutational status was only determined in a subset of 12 patients, in whom *KIT* exon 11 mutations were demonstrated in 10, *KIT* exon 9 mutation in 1, and no mutation in 1. In this retrospective study, tumor mutational testing for the oncogenic *KIT* and/or *PDGFRA* genes was able to be performed in only 12 of 41 (29%) of these rare patients with rectal GISTs. Given the known challenges described below, obtaining evaluable tissues from patients identified over more than a decade is simply not feasible. In three patients who underwent resection of the primary tumor between 2000 and 2008, paraffin-embedded blocks were collected, but the quality of tumor material was insufficient to perform mutational testing because of technical challenges associated with these older blocks and the tissue fixation procedure. For nine other patients, resection of primary GISTs was performed before 1999, and the quality of the tissue specimen from the primary tumor was insufficient to determine the mutational status. Thus, despite our best efforts, retrospective mutational examination of any additional patients in our series is unfortunately not possible. However, our team believes that the information provided in this series is useful because tumor mutational testing is routinely performed in only a minority (approximately 5%) of GISTs worldwide. Nevertheless, the mutation frequency in rectal GISTs has not been specifically investigated [15,16] except in Miettinen’s study [8], which found mutations in 18 of 29 (62%) of the cases evaluated, with a predominance of *KIT* exon 11 mutations, a lack of *KIT* exon 13 mutations, and only one *KIT* exon 9 mutation.

In our study, 56% of the patients were classified in the high-risk group according to the Miettinen classification [19], and 56% of patients relapsed after surgical resection of primary localized GISTs with disease-free survival rates of 53.9 and 34.6% at 3 and 5 years, respectively. This confirms the more aggressive behavior of rectal GISTs. These data also corroborated the results of Mussi et al. [21], Tsai et al. [24], and Miettinen et al. [8], who showed that the majority of rectal GISTs are large, demonstrate high-risk malignant behavior, and have a tendency to recur and metastasize. Furthermore, in three Korean studies, colorectal GISTs occurred predominantly in the rectum and tended to be classified as high-risk, which was the most important risk factor for recurrence [25-27].

The cornerstone in treatment for patients with localized GISTs is complete surgical resection with *en bloc* tumor removal and clear margins, avoiding tumor rupture. In our series, the relatively high rate of tumor rupture during surgical resection might be explained by several factors in this rare subset of patients with GIST. In particular, this is a particularly challenging anatomical location for primary GISTs, and the risk of rupture definitely seems higher than that in many other sites. In five of our patients, the large size of the primary tumor (8–10 cm) in this constrained anatomic space likely contributed to the risk of rupture. In another three patients, the surgical resection was performed by endoanal or transvaginal enucleation with resultant fragmentation of the tumor. Conversely, rectal GISTs are often large tumors. Therefore, they are a challenge for surgeons because of the confined pelvic space (anatomical location close to the anal sphincters, pelvic nerves, and bladder) and the fact that they are often adherent to the pelvic floor. As a consequence, rectal GISTs might require extensive surgery (e.g., abdominoperineal or multivisceral resection) to achieve complete surgical resection. Nevertheless, various conservative surgical procedures and approaches may be considered for rectal GISTs, including local excision, anterior resection, trans-sacral/anal/vaginal approaches, and laparoscopic approaches [28-30]. The choice of the procedure mainly depends on the tumor size and location in relation to the anorectal margin. In our series, only 11 (27%) patients underwent abdominoperineal resection, and 30 patients were eligible for conservative surgery with various surgical procedures.

Nevertheless, resections with clear margins (R0 resections) were more common with abdominoperineal procedures than with conservative surgery (8/11 and 14/30, respectively), but tumor recurrence occurred similarly in both groups of patients: in 6 of 11 patients (54%) after abdominoperineal surgery and in 11 of 30 patients (46.6%) after conservative surgery.

Because of the rarity of this disease, no prospective studies have compared radical surgery versus conservative surgery or local tumor excision, and only such a study could elucidate the value of this radical surgery. Khalifa et al. [31] reported that there was no difference in survival rates between local resection and abdominoperineal resection in patients with rectal sarcoma. We believe that local resection should be performed if microscopically clear margins can be safely achieved.

Preoperative imatinib therapy was administered to 12 (30%) of the patients in this series because of large GISTs, difficulty of complete tumor removal, and preservation of the anal sphincters. In all cases, it enabled a modification in tumor size and/or density. It also permitted the performance of conservative surgery in 8 of 12 patients (6 of these 8 tumors were located in the lower third of the rectum). Thus, this treatment was feasible, safe, and effective. Since 2005, several case reports or small series regarding the use of preoperative imatinib treatment for rectal GISTs have been published [10-13,32-35]. All concluded that preoperative imatinib therapy has an important role in downsizing large rectal GISTs and in reducing the mitotic activity. Because these tumors are in the vicinity of pelvic structures (i.e., bladder, major pelvic nerves, and anal sphincters) and given that radical surgery may lead to considerable morbidity, downstaging might be beneficial in this situation, allowing function-sparing procedures and less invasive surgery while potentially improving tumor resectability. Preoperative treatment is thus a reasonable option for patients with locally advanced rectal GISTs that require abdominoperineal or multivisceral resection for complete tumor removal.

Controversy remains regarding the optimal duration of preoperative therapy. Our patients received preoperative imatinib for a median duration of 7 months (range, 2–10 months). In the EORTC phase III trial, the median time to best response was 4 months, but some responses were documented later [36]. Similar observations have been made in case reports of preoperative imatinib in localized diseases [37]. Therefore, it would be reasonable to plan a final surgery within 6 to 12 months of imatinib onset [34].

Interestingly in our series, 18 patients (72%) in the non-imatinib group and only 2 (16%) in the imatinib group developed local recurrence. We showed that preoperative and/or postoperative imatinib significantly reduced the risk of not only local recurrence (p = 0.006), but also overall relapse (p = 0.011), and significantly improved the disease-free survival with no impact on overall survival. These results are in accordance with those from Andtbacka et al. [38] on 16 locally advanced GISTs from several locations treated with preoperative imatinib and those from Tielen et al. on 32 rectal GISTs, 22 treated with preoperative imatinib and 10 with surgery only [15].

## Conclusions

Based on these data, we suggest that a therapeutic strategy combining surgery with preoperative imatinib therapy should be systematically considered for patients with rectal GISTs, specifically for patients with larger tumors, marginally resectable tumors, or tumors close to the anal sphincters because imatinib may potentially increase the proportion of patients able to undergo conservative surgery rather than the more morbid abdominoperineal resection. Furthermore, according to the results of the ACOSOG Z9001 study and two recent Scandinavian studies [17,39], the indication to continue imatinib in the postoperative setting for a duration of at least 1 year has been established in patients with high-risk GISTs, when incomplete surgical resection has occurred, or when tumor rupture has occurred. This should be systematically discussed with patients according to the risk of disease recurrence per the standard risk assessment criteria.

Due to the rarity of these tumors, these decisions require the multidisciplinary expertise of surgeons, medical oncologists, radiologists, and gastroenterologists in expert centers. Referral of patients with this rare disease to expert centers is recommended.

## Competing interests

The authors declare that they have no competing interests.

## Authors’ contributions

TKH + FD have made substantial contributions to conception and design. JM participated in the design of the study and performed the statistical analysis. All the authors have been involved in drafting the manuscript and revising it critically for important intellectual content. All the authors have given final approval of the version to be published.

## Pre-publication history

The pre-publication history for this paper can be accessed here:

http://www.biomedcentral.com/1471-2407/14/156/prepub
